# What “Family Affair?” Domestic Violence Awareness in China

**DOI:** 10.3389/fpubh.2022.795841

**Published:** 2022-03-04

**Authors:** Zhaohui Su, Dean McDonnell, Ali Cheshmehzangi, Junaid Ahmad, Hengcai Chen, Sabina Šegalo, Yuyang Cai

**Affiliations:** ^1^School of Public Health, Institute for Human Rights, Southeast University, Nanjing, China; ^2^Department of Humanities, South East Technological University, Carlow, Ireland; ^3^Faculty of Science and Engineering, University of Nottingham Ningbo China, Ningbo, China; ^4^Network for Education and Research on Peace and Sustainability (NERPS), Hiroshima University, Hiroshima, Japan; ^5^Prime Institute of Public Health, Peshawar Medical College, Peshawar, Pakistan; ^6^Department of Microbiology, Faculty of Medicine, University of Sarajevo, Sarajevo, Bosnia and Herzegovina; ^7^School of Public Health, Shanghai Jiao Tong University School of Medicine, Shanghai, China; ^8^China Institute for Urban Governance, Shanghai Jiao Tong University, Shanghai, China

**Keywords:** domestic violence, COVID-19, china, family affairs, public health, interventions

## Abstract

**Introduction:**

Domestic violence is toxic to society. With approximately one in three women on average falling victim to domestic violence, systematic solutions are needed. To further complicate the issue, mounting research shows that COVID-19 has further exacerbated domestic violence across the world. Situations could be even more pronounced in countries like China, where though domestic violence is prevalent, there is a dearth of research, such as intervention studies, to address the issue. This study investigates key barriers to domestic violence research development in China, with a close focus on salient cultural influences.

**Methods:**

A review of the literature on domestic violence in China in PubMed, PsycINFO, and Scopus was conducted to answer the research question. The search was focused on three themes, domestic violence, China, research, and cultural influences.

**Results:**

The study findings show that categorizing domestic violence as a “family affair” is a key barrier to domestic violence research development in China—an incremental hindrance that prevents the public and policymakers from understanding the full scale and scope of domestic violence in China. In addition to abusers, witnesses, and victims, even law enforcement in China often dismisses domestic violence crimes as “family affairs” that resides outside the reach and realm of the law. The results indicated that mistreating domestic violence crimes as “family affairs” is a vital manifestation of the deep-rooted cultural influences in China, ranging from traditional Confucian beliefs in social harmony to the assumed social norms of not interfering with other people's businesses.

**Conclusion:**

Domestic violence corrupts public health and social stability. Our study found that dismissing domestic violence cases as “family affairs” is an incremental reason why China's domestic violence research is scarce and awareness is low. In light of the government's voiced support for women's rights, we call for the Chinese government to develop effective interventions to timely and effectively address the domestic violence epidemic in China.

## Introduction

Domestic violence is toxic to society. Domestic violence or violence against women could be understood as “any act of gender-based violence that results in, or is likely to result in, physical, sexual, or mental harm or suffering to women, including threats of such acts, coercion or arbitrary deprivation of liberty, whether occurring in public or in private life” (1). Domestic violence not only has a devastating effect on personal and public health, it is also a constant threat to social stability and global solidarity (2). On an individual level, mounting research shows that domestic violence could result in long-term damages to people's physical and psychological health (3–8). On a global level, data from the World Health Organization show that one in every three women falls victim to domestic violence (2).

However, due to a lack of research, there is a shortage of up-to-date and systematic investigations of domestic violence in developing countries. What is clear, though, is that currently available research paints a dire picture. A systematic review study that pooled all published papers conducted in low- and middle-income countries indicates that, for instance, lifetime intimate partner violence against women was as prevalent as 55% (9). Additional compounding factors are also present. As seen amid the pandemic, partially due to issues such as prolonged time spent with the abusers, deteriorated financial stability, and disrupted local and national support programs, COVID-19 has further exacerbated domestic violence worldwide (10–12). In the early days of the pandemic, for instance, police records show that in Jianli city, Hubei Province, China, there was a three-fold hike of domestic violence cases influenced heavily by the pandemic and its prevention measures (e.g., lockdowns) (13).

While similar trends are also present in developed countries, what is unique about domestic violence in countries like China is that the victims often face the dual burden of lack of systematic awareness at the societal level (i.e., dearth of timely and quality research) and have limited timely and systematic support from society. Traditionally, a key solution to address domestic violence has been via effective intervention programs, ranging from researchers-led programs, non-profit organizations initiated support groups, local government-sponsored hotlines, or state-funded safe houses (14). However, these interventions are often poorly developed in countries such as China (15), where there is a pronounced dearth of research on domestic violence and a lack of solutions to which victims could turn for help. In other words, though domestic violence's prevalence in China has long been established —similar if not worse than the global average (i.e., 34%) (16), domestic violence research and interventions in China are often rudimentary and of suboptimal priority (17).

This lack of priority is also reflected in police officers' attitudes toward domestic violence cases in China. In a study on 623 police officers in China, for instance, researchers found that nearly half of law enforcement personnel (i.e., 46.5%) are likely to not make arrests of domestic violence abusers, regardless of the severity of the violence (18). These insights combined, overall, further underscore the imperative for in-depth and comprehensive research on domestic violence in China, which in turn, could raise the awareness of domestic violence across society and help government and health officials to develop evidence-based intervention programs to eliminate, if not eradicate, domestic violence in China. However, to date, there is a dearth of investigations on factors that negatively influence the research development on domestic violence in China, particularly in light of the unique insights exposed by the COVID-19 pandemic (15). Thus, to bridge the research gaps, this study aims to examine the key barriers to domestic violence research development in China, with a close focus on salient cultural influences.

## Methods

A review of the literature on domestic violence research in China in PubMed, PsycINFO, and Scopus was conducted to answer the study question. In the context of this study, barriers to domestic violence research development in China were operationalized as incremental links that prevent the public and the policymakers from understanding the scale and scope of domestic violence in China. While a number of cultural influences and social norms could shape domestic violence in China, we only focused on the most salient factors (e.g., most cited) identified in academic literature. The narrative literature review approach was adopted as the review framework, which could be understood as “an objective, thorough summary and critical analysis of the relevant available research and non-research literature on the topic being studied” (19). One key advantage of the narrative literature review approach is that it could help the researchers gain a structured understanding of the literature in an effective manner (19).

**Figure 1:**
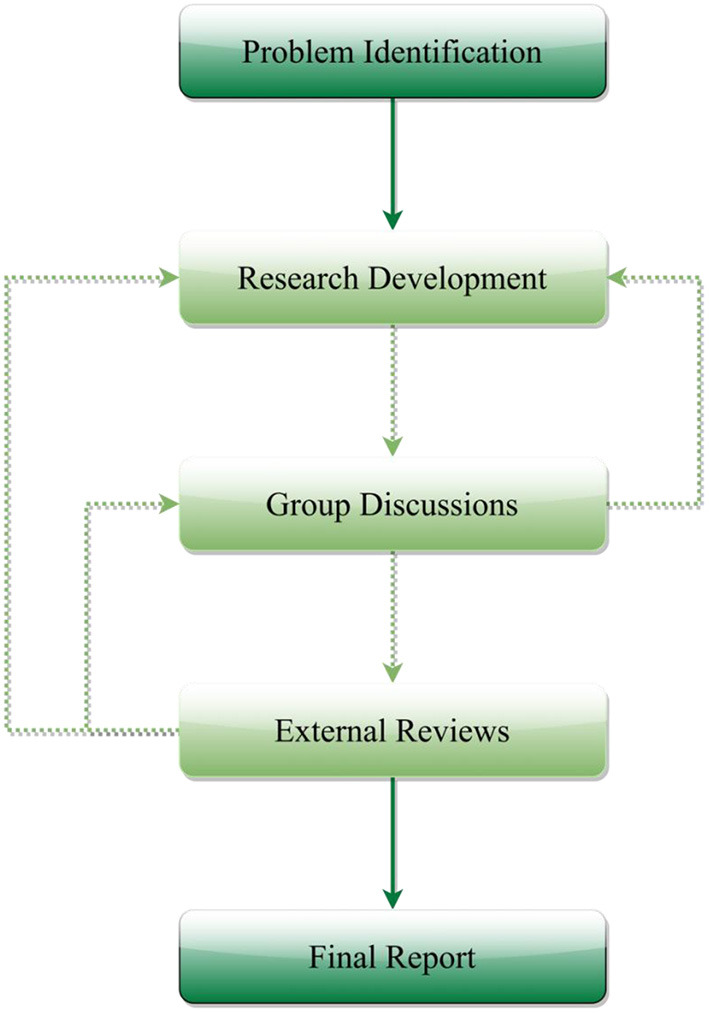
A schematic representation of the research flow.

The search was focused on four themes, domestic violence, China, research, and cultural influences. Though domestic violence could happen to both men and women (20), as women are often the predominant gender in domestic violence victims, our study focused on female domestic violence. An example PubMed search term could be found in Table 1. The search was first conducted on May 25, 2021, with the follow-up review completed on December 28, 2021. A schematic representation of the research flow could be found in Figure 1. Scholarly papers were excluded if they: (1) were not published in English, (2) were not focused on domestic violence research on women, and (3) could not provide key insights on the influences of cultural or social norms on domestic violence in China. To ensure up-to-date insights could be considered to help contextualize the findings and white papers were also reviewed and analyzed.

**Table 1:** Example PubMed search strings.

**Theme**	**Search string**
Domestic violence	“Domestic violence”[MeSH] OR “domestic violence”[TIAB] OR “intimate partner violence”[MeSH] OR “intimate partner violence”[TIAB] OR “family violence” OR “violence against women”
Cultural influences	Culture*[MeSH] OR culture*[TIAB] OR attitude*[MeSH] OR attitude*[TIAB] OR norm*[MeSH] OR norm*[TIAB] OR “social norm*” OR “cultural influence*” OR belief*
Research	Research[MeSH] OR Research[TIAB] OR investigation* OR studies OR study OR intervention*
China	China[MeSH] OR China[TIAB] OR Chinese[MeSH] OR Chinese[TIAB]

## Results

Of the total number of articles included in the review and subsequent analysis (*N* = 21), the vast majority were published within the last 5 years (*n* = 12), and adopted various methodological approaches. Some of the reviewed studies used qualitative approaches investigated domestic violence in the context of family experiences [e.g., (21)] or a series of interviews with males (*n* = 18) who had used violence against their partner [e.g., (22)]; while others adopted more quantitative approaches, such as cross-sectional designs with large groups of participants [e.g., (23, 24)], or a pre-post experimental design with police officers (N=401) in evaluating changing attitudes toward domestic violence [e.g., (25)]. Detailed information on the list of articles reviewed could be found in Table 2.

**Table 2:** List of articles reviewed.

**Author**	**Year**	**Title**
Chan (22)	2006	The Chinese concept of face and violence against women
Ko Ling et al. (23)	2008	Understanding violence against Chinese women in Hong Kong: an analysis of risk factors with a special emphasis on the role of in-law conflict
Ko Ling (26)	2009	Sexual violence against women and children in Chinese societies
Hayes et al. (25)	2020	Chinese police cadets' attitudes toward domestic violence: a pretest/posttest design
He and Hang Ng (27)	2013	In the name of harmony: the erasure of domestic violence in China's judicial mediation
Jiang (28)	2019	The family as a stronghold of state stability: two contradictions in China's anti-domestic violence efforts
Li et al. (29)	2021	Tolerance for domestic violence: do legislation and organizational support affect police view on family violence?
Lin et al. (18)	2020	Chinese police officers' attitudes toward domestic violence interventions: do training and knowledge of the Anti-Domestic Violence Law matter?
Lin et al. (30)	2021	Rank, experience, and attitudes toward domestic violence intervention: a moderated mediation analysis of Chinese police officers
Lu and Hao (31)	2021	Combating domestic violence during COVID-19: what does the Chinese experience show us?
Qi et al. (32)	2020	Anti-domestic violence law: the fight for women's legal rights in China
Qu et al. (33)	2018	Correlates of attitudes toward dating violence among police cadets in china
Sun et al. (34)	2020	Officer and organizational correlates with police interventions in domestic violence in China
Tsun and Lui-Tsang (21)	2005	Violence against wives and children in Hong Kong
Xie et al. (35)	2017	Domestic violence counseling in rural northern China: gender, social harmony, and human rights
Xu et al. (36)	2020	Empirical study on handling of domestic violence cases by police, China Journal of Social Work
Xue (37)	2008	Perceptions of and attitudes toward domestic violence in China: implications for prevention and intervention
Wang et al. (38)	2019	Correlates of Chinese police officer decision-making in cases of domestic violence
Wang et al. (24)	2021	Officers' preferences for gender-based responding to domestic violence in China
Wu et al. (39)	2020	Organizational support and Chinese police officers' attitudes toward intervention into domestic violence
Zhao et al. (40)	2018	The tendency to make arrests in domestic violence: perceptions from police officers in China

The study findings show that categorizing domestic violence as a “family affair” is a key barrier to domestic violence research development in China—an incremental hindrance that prevents the public and policymakers from understanding the full scale and scope of domestic violence in China. In addition to abusers, witnesses, and victims, even law enforcement in China often dismisses domestic violence crimes as “family affairs” that reside outside the reach and realm of the law [e.g., (36)]. The results indicated that mistreating domestic violence crimes as “family affairs” is a vital manifestation of the deep-rooted cultural influences in China, ranging from traditional Confucian beliefs in social harmony to the assumed social norms of not interfering with other people's businesses [e.g., (27)]. A list of exemplary quotes could be found in Table 3.

**Table 3:** Example quotes from the articles reviewed.

**Author**	**Year**	**Quote**
Cao et al. (41)	2016	“Domestic violence in China is traditionally regarded as a **family affair**, thus it is beyond social and governmental concern and beyond the consideration of Chinese modern law”.
Chen et al. (42)	2021	“For a longtime, domestic violence has only been regarded as a **family affair**, protected by family privacy and other traditional values; and as a result, it is beyond the social and governmental concern, and beyond the coverage of the modern law until 2016”.
Chung et al. (43)	1996	“Moreover, most Chinese believe that a **family affair** is a private matter and other people should not intervene. Some nurses may hold these traditional beliefs and attitudes and this may affect their approach and care to battered women”.
Jiang (28)	2019	“In more severe cases, a lot of relatives and friends will show up and take part, trying all-out efforts or even cajole the wives to withdraw their domestic violence reports by brainwashing her with the cliché that ‘every couple will fight and quarrel; it is shameful to make **family affairs** public.’ Eventually, the wives surrender”.
Lau and Chan (44)	2008	“Chinese people with traditional beliefs tend to believe that a family problem is private **family affair** (jia shi) that should not be handled by people”.
Li et al. (45)	2020	“Furthermore, domestic violence is considered as a private, often shameful, **family affair** that should not be disclosed to outsiders”
Liu et al. (46)	2018	“On the basis of a series of researches and practice in preventing from IPV [intimate partner violence] in China, a new law on IPV has taken effect. Under the law, IPV is no longer considered a “**family affair**,” but a legal issue that demands action from the courts and the police”.
Loke et al. (47)	2012	“However, when health professionals hold the cultural belief that **family affairs** are a private matter, this may affect their approach toward women who suffer from intimate partner violence. Even if women seek health care, most people, including nurses, still believe that IPV is a private family matter in which other people should not intervene”.
Lu and Hao (31)	2021	“In Chinese culture, domestic violence was once considered a private **family affair** that should not be regulated by the public power”.
Qi et al. (32)	2020	“Due to the long-term influence of this traditional system that has emphasized family stability in China, domestic violence has often been tolerated or acquiesced to by government and society, who tend to classify its incidence as an internal **family affair**”.
Xu et al. (36)	2020	“Officers who took part in the interviews no longer think that DV was only a **family affair** and generally declared all the cases involving DV would be handled immediately; after the information from residents' committees about DV sent to the officers, community police officers would also contact with the related persons immediately”.
Yue et al. (48)	2019	“The main character Mei Xiangnan is a school teacher who endured severe physical and psychological abuse by her husband An Jiahe, a well-respected surgeon. Mei silently tolerated the domestic abuse out of fear that she would be ostracized if she made public what was a private **family affair**. Instead of ebbing, the cycle of violence kept escalating”.

## Discussion

This study set out to investigate key barriers to domestic violence research development in China, with a close focus on salient cultural influences. Our research is the first that explored barriers that hinder the public and policymakers' understanding of the full scale and scope of domestic violence in China. While many factors shape the lack of domestic violence research in China, we focused on salient cultural influences that are most discussed in published academic papers. The study findings show that, by diminishing domestic violence crimes into “private” “family affairs”, cultural and social norms play a defining role in shaping the domestic violence narrative and public awareness of the topic in China (27). The results also indicate that almost all key stakeholders, ranging from abusers, witnesses, families and friends, victims, to police officers, often dismiss domestic violence crimes as matters of “family affairs” that reside outside the reach and realm of the law [e.g., (24, 28, 47)]. These sobering insights combined, in turn, shed light on why the current research development is insufficient to inform the public and policymakers about the full scale and scope of domestic violence in China.

### Domestic Violence as a “Family Affair”

Our findings show that categorizing domestic violence as a “family affair” is a critical barrier to domestic violence research development in China—an incremental hindrance that prevents the public and policymakers from understanding the prevalence, severity, and repercussions of domestic violence in China. In light of the human and economic tolls associated with domestic violence, the public nature of domestic violence crimes, as indicated in its impacts on all sectors of societies, as opposed to limiting it as “private” and “family affairs”, is abundantly evident (49–53). Analyses conducted in the United States in 2014, for instance, show that domestic violence lifetime cost was $103,767 per female victim and $23,414 for individual male victims, paired with the fact that 43 million U.S. adults are victims of domestic violence, the total cost of domestic violence on society could amount to $3.6 trillion over victims' lifetime (54). However, due to poor research development, it is unclear in terms of what might be the totality of the human and economic toll of domestic violence in China, let alone insights that factor in the most up-to-date impacts of COVID-19 on the scale, scope, and severity of domestic violence in the country.

What is clear, though, is China has nearly four times the U.S.'s population (55), and lacks the domestic violence prevention infrastructure that is available in developed countries such as the U.S. (56–58), critical intervention mechanisms that have the potential to help victims fend off the adverse impacts of domestic violence. These insights combined, in turn, further underscore the problematic nature of the research gap. Without essentials such as domestic violence crime reporting, monitoring, and surveillance, basic understanding such as what are the impacts of domestic violence on avoidable medical experiences, workforce productivity lost, and long-term social stability could not be achieved. Needless to say, without these understandings, government and health officials may not be able to evaluate, let alone prevent, the repercussions of domestic violence as an overlooked public health epidemic on the health and wellbeing of the society at large.

**Figure 2:**
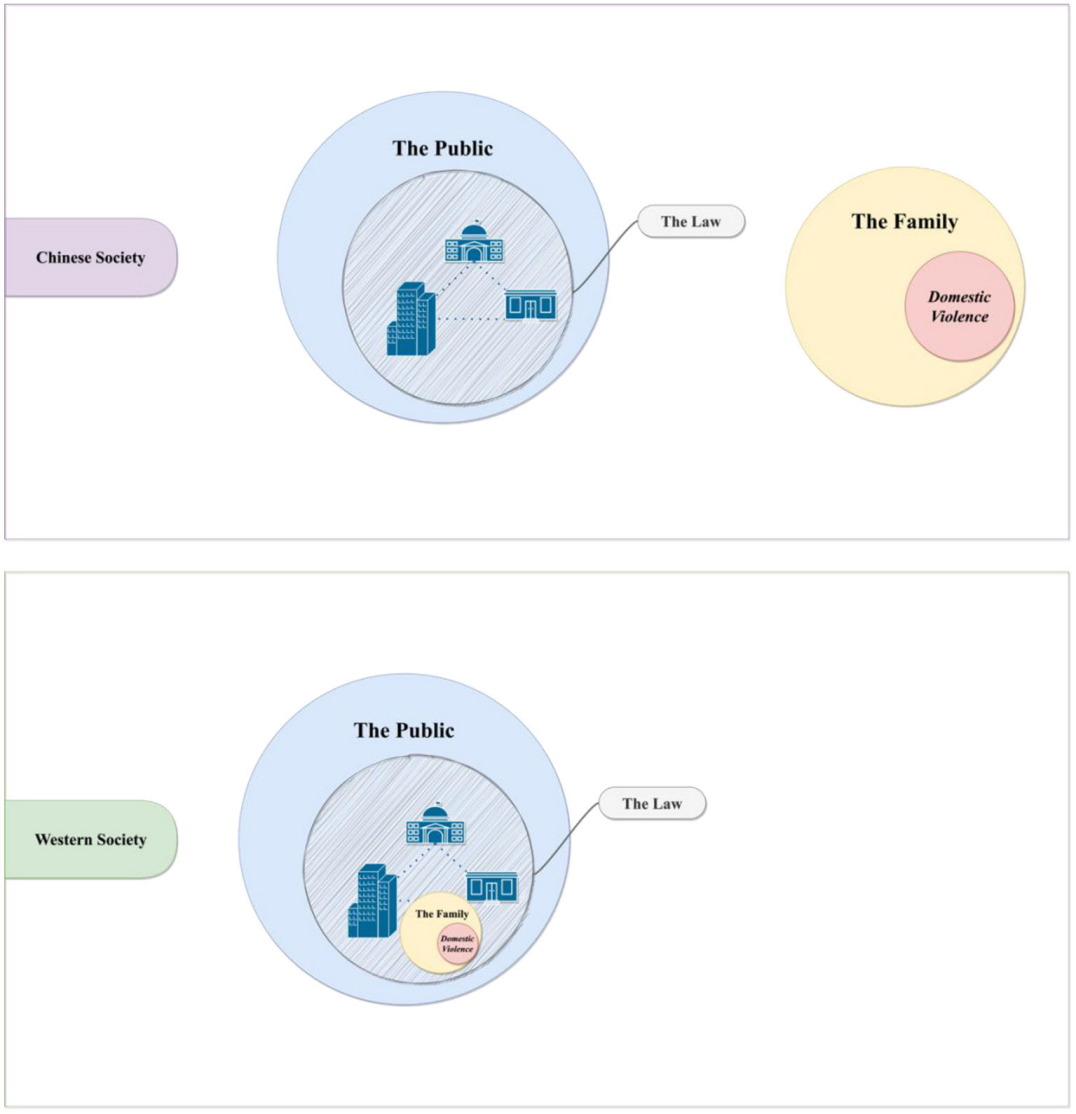
A schematic representation of the unique categorization of domestic violence in China.

It is important to note that dismissing domestic violence crimes as matters of “family affairs” is not a phenomenon that is unique to China. Many societies, for instance, either due to population makeup or geographical proximity to China, also face the issue (59–63). Furthermore, cultures that are often influenced by traditional or conventional social beliefs, such as the Arab world, also found that “family affairs” could be a frequent excuse cited by abusers for their crimes and a salient barrier that prevents victims from seeking help (64–66). However, what is unique about categorizing domestic violence crimes as “family affairs” in China centers on its prevalence and pervasiveness. The study findings show that, in addition to abusers, witnesses, and victims, even law enforcement in China often dismisses domestic violence crimes as “family affairs” that reside outside the reach and realm of the law (40, 45, 67). A schematic representation of the unique categorization of domestic violence in Chinese society, as found in our analyses, could be seen in Figure 2.

In a study on accident and emergency nurses in Hong Kong, researchers found that 43% of the participants do not believe they have a duty to intervene in violence domestic cases (43). What is equally, if not more, alarming, is that in the same study, among all of the 57% of nurses who indicated they should intervene, all of them endorsed the traditional Chinese saying— “even a good judge cannot adjudicate family disputes” (43). Similar patterns—failing to help domestic violence victims per their job descriptions and the requirement of the law—are also found in other stakeholders (47). These insights combined suggest that in order to fundamentally change this corrosive practice of labeling domestic violence crimes as “family affairs”, systematical measures led by government and health officials are needed urgently.

### A Social Ecological Approach

One way to effectively change the cultural influences and social norms associated with equating domestic violence as a “family affair” is via addressing the issue from a social ecological perspective (68). The social ecological model suggests that public behaviors are often shaped by various influences with different levels of social implications. These influences could often be grouped into the intrapersonal, interpersonal, organizational, community, and policy level factors (68). Drawing insights from the social ecological model, we propose a government-led, community-facilitated, organization-sponsored, and individual-responsible approach to address the deep-rooted cultural and social influences on domestic violence in China.

### Change the “Family Affair” Narrative

It is important to underscore that, as evidenced in behavioral sciences literature (69–71), referencing domestic violence as a “family affair” could reduce the perceived severity and create a climate of complacency in society. To effectively address this issue, media professionals, health organizations, and perhaps most importantly, government agencies need to take a more significant role in changing the narrative surrounding domestic violence. Government agencies, for instance, especially those involved in or associated with law enforcement, need to set the example of banning the misleading use of “family affair” in formal and informal reference to domestic violence. Persuasive health campaign interventions could also help change the narrative. Health campaign interventions can be understood as using communication or marketing tools and techniques to change the target audience's attitudes and behavior toward a health phenomenon (72). As the literature suggests, health campaign interventions are effective in shifting culture and social norms around a social phenomenon (72–76). Considering that the association between domestic violence and “family affair” is deeply rooted in the Chinese culture, the government must initiate health campaign interventions tailored to social norms around the “family affair” phenomenon (77–79).

### Improve Women's Financial Autonomy

It is important to note that the Chinese government has been making progress in improving women's rights. In a Lancet study on Chinese women's overall health and quality of life development, ample evidence shows that substantial strides were made in the past 70 years (80). However, ingrained issues may need the government's sustained intervention to address. For instance, though China's gender wage gap has been relatively low compared to countries such as the U.S. (81), it has been on the rise (82). Recurring evidence shows that financial capabilities are often closely linked to women's susceptibility to domestic violence (83). In other words, it is possible that financial autonomy has the potential to help women think and live above submissive culture patterns (84), such as cultural influences that translate domestic violence crimes into “family affairs”. These insights combined suggest that addressing the gender wage gap in China could be a key strategy that the Chinese government could adopt to mitigate domestic violence.

### Interventions That Hold Individuals Responsible

Another critical intervention that policymakers should consider is improving the utility and functionality of the 2016 Anti-Domestic Violence Law in China, implementation of which has been lax among law enforcement personnel (32, 85, 86). In addition to traditional intervention programs designed for abusers (e.g., training or rehabilitation opportunities) (87), another way to address this issue is via incorporating domestic violence abuser history for the past 5 years as a part of individuals'—both male and female job seekers—eligibility for applying for and working at the public sectors. In other words, people with a record of domestic violence crimes for the past 5 years should not be allowed to apply for working as public servants, vital personnel who might be exposed to tasks that are inappropriate for abusers, such as domestic violence victims relocation.

A key advantage of this program centers on its non-judgmental attitude toward whether domestic violence crimes are “family affairs”. Essentially, the proposed intervention approach aims to address domestic violence at the behavioral level, as opposed to the belief level—regardless of which belief systems the abusers hold or how conventional their views toward family or women might be (88–90), if they commit domestic violence crimes for the past 5 years, they should not be allowed in desirable and high-stake jobs like pubic servants. Needless to say, due to its novelty—no such interventions have been previously deployed in China, rigorous and evidence-based development processes are needed to ensure this intervention has the potential to reach the desirable intervention outcome—reduce domestic violence prevalence in China in scale. In the same vein, the central government should also make abilities to implement the Anti-Domestic Violence Law a key capacity of the local governments. As a matter of fact, Changsha, the capital city of Hunan Province, China, has already made domestic violence prevention a key component of their performance assessment rubric—effectively making abilities to prevent domestic violence a part of public officials' job descriptions (91). Overall, the promises of these approaches center on their potential to incentivize both individuals and local governments to invest in domestic violence prevention efforts on a daily basis.

### Community-Facilitated Collaborative Interventions

In addition to incentivizing current or formal domestic violence abusers to not commit domestic violence crimes via potential career prospects and job opportunities, limiting these perpetrators' access to victims or opportunities to inflict harm may also be a practical approach. For instance, making domestic violence history searchable in the national marriage registry free of charge could help women avoid being exposed to these abusers. To further incentivize abusers to refrain from harming their current or formal intimate partners, making the records “dormant” if the perpetrators are able to manage to not commit domestic violence for 5 years could be another effective behavior nudge. Currently, Yiwu, a southern city in China, has already made such a searchable digital system available city-wide (92). Overall, this big data-based domestic violence surveillance and monitoring mechanism have the potential to engage all stakeholders, ranging from victims, families and friends, community members, city officials, health experts, to abusers, in curbing and controlling domestic violence crimes across sectors of societies.

### Limitations

While our study bridges important research gaps, it is not without limitations. First, the narrative review approach was adopted to gauge the research question. While a narrative review could be a key step in determining the suitability of systematic reviews, the non-systematic nature of the research method nonetheless limits the study results' reproducibility and replicability (93). Furthermore, we only reviewed scholarly literature published in English. As our key research objective is to gauge the scale and scope of academic work available on domestic violence in China, this decision is well-justified. However, by doing this, it is possible that insights published in Chinese or other languages in non-academic venues are not presented in this study. To address these issues, future research could adopt the systematic review approach to examine the research question with a search that is both broad and comprehensive.

## Conclusion

Domestic violence endangers public health and social stability. Our study found that dismissing domestic violence crimes as “family affairs” is a key reason why China's domestic violence research is scarce and awareness is low. In light of the government's voiced support for women's rights, we call for the Chinese government to develop effective interventions to timely and effectively address the domestic violence epidemic in China. To further shed light on the issue, future research could develop interventions that target the corrosive practice of treating domestic violence in China as a “family affair” to further improve women's health and quality of life, public health and wellbeing, social stability, as well as global solidarity.

## Author Contributions

ZS developed the research idea and drafted the manuscript. DM, AC, JA, HC, SŠ, and YC reviewed and revised the manuscript. All authors contributed to the article and approved the submitted version.

## Funding

This work was supported by the Institute of College Student Development, Shanghai Jiao Tong University (grant number DFY-SJ-2021055).

## Conflict of Interest

The authors declare that the research was conducted in the absence of any commercial or financial relationships that could be construed as a potential conflict of interest.

## Publisher's Note

All claims expressed in this article are solely those of the authors and do not necessarily represent those of their affiliated organizations, or those of the publisher, the editors and the reviewers. Any product that may be evaluated in this article, or claim that may be made by its manufacturer, is not guaranteed or endorsed by the publisher.
