# Corrigendum: What “Family Affair?” domestic violence awareness in China

**DOI:** 10.3389/fpubh.2022.990348

**Published:** 2022-07-27

**Authors:** Zhaohui Su, Dean McDonnell, Ali Cheshmehzangi, Junaid Ahmad, Hengcai Chen, Sabina Šegalo, Yuyang Cai

**Affiliations:** ^1^Center on Smart and Connected Health Technologies, Mays Cancer Center, School of Nursing, UT Health San Antonio, San Antonio, TX, United States; ^2^Department of Humanities, Institute of Technology Carlow, Carlow, Ireland; ^3^Faculty of Science and Engineering, University of Nottingham Ningbo China, Ningbo, China; ^4^Network for Education and Research on Peace and Sustainability (NERPS), Hiroshima University, Hiroshima, Japan; ^5^Prime Institute of Public Health, Peshawar Medical College, Peshawar, Pakistan; ^6^Department of Microbiology, Faculty of Medicine, University of Sarajevo, Sarajevo, Bosnia and Herzegovina; ^7^School of Public Health, Shanghai Jiao Tong University School of Medicine, Shanghai, China; ^8^China Institute for Urban Governance, Shanghai Jiao Tong University, Shanghai, China

**Keywords:** domestic violence, COVID-19, china, family affairs, public health, interventions

In the published article, there was an error in the Funding statement. The grant number was incorrectly written as “DFY-LL-2020081.” The correct Funding statement is: “This work was supported by the Institute of College Student Development, Shanghai Jiao Tong University (grant number DFY-SJ-2021055).”

The authors apologize for this error and state that this does not change the scientific conclusions of the article in any way. The original article has been updated.

## Publisher's note

All claims expressed in this article are solely those of the authors and do not necessarily represent those of their affiliated organizations, or those of the publisher, the editors and the reviewers. Any product that may be evaluated in this article, or claim that may be made by its manufacturer, is not guaranteed or endorsed by the publisher.

