# Corrigendum: What “family affair?” domestic violence awareness in China

**DOI:** 10.3389/fpubh.2022.1122931

**Published:** 2023-01-04

**Authors:** Zhaohui Su, Dean McDonnell, Ali Cheshmehzangi, Junaid Ahmad, Hengcai Chen, Sabina Šegalo, Yuyang Cai

**Affiliations:** ^1^School of Public Health, Institute for Human Rights, Southeast University, Nanjing, China; ^2^Department of Humanities, South East Technological University, Carlow, Ireland; ^3^Faculty of Science and Engineering, University of Nottingham Ningbo China, Ningbo, China; ^4^Network for Education and Research on Peace and Sustainability (NERPS), Hiroshima University, Hiroshima, Japan; ^5^Prime Institute of Public Health, Peshawar Medical College, Peshawar, Pakistan; ^6^Department of Microbiology, Faculty of Medicine, University of Sarajevo, Sarajevo, Bosnia and Herzegovina; ^7^School of Public Health, Shanghai Jiao Tong University School of Medicine, Shanghai, China; ^8^China Institute for Urban Governance, Shanghai Jiao Tong University, Shanghai, China

**Keywords:** domestic violence, COVID-19, China, family affairs, public health, interventions

In the published article, there were errors in the affiliations of Zhaohui Su and Dean McDonnell.

Instead of “Center on Smart and Connected Health Technologies, Mays Cancer Center, School of Nursing, UT Health San Antonio, San Antonio, TX, United States,” the affiliation of Zhaohui Su should be “School of Public Health, Institute for Human Rights, Southeast University, Nanjing, China.”

There was an error in affiliation 2. Instead of “Institute of Technology Carlow” it should read “South East Technological University.” The full corrected affiliation of Dean McDonnell is “Department of Humanities, South East Technological University, Carlow, Ireland.”

The authors apologize for these errors and state that this does not change the scientific conclusions of the article in any way. The original article has been updated.

